# Genetic Diversity of Salp15 in the *Ixodes ricinus* Complex (Acari: Ixodidae)

**DOI:** 10.1371/journal.pone.0094131

**Published:** 2014-04-08

**Authors:** Xin Wang, Yong Huang, Si-bo Niu, Bao-Gui Jiang, Na Jia, Leo van der Geest, Xue-bing Ni, Yi Sun, Wu-Chun Cao

**Affiliations:** 1 State Key Laboratory of Pathogen and Biosecurity, Beijing Institute of Microbiology and Epidemiology, Beijing, P. R. China; 2 Wenzhou Center for Disease Control and Prevention, Wenzhou, P. R. China; 3 State Key Laboratory of Veterinary Biotechnology, Harbin Veterinary Research Institute, Chinese Academy of Agricultural Sciences, Harbin, P. R. China; 4 Institute for Biodiversity and Ecosystem Dynamics, Section Population Ecology, The University of Amsterdam, Amsterdam, Netherlands; University of Kentucky College of Medicine, United States of America

## Abstract

Salp15, a 15-kDa tick salivary gland protein, is both essential for ticks to successfully obtain host blood and also facilitates transmission of Lyme borreliosis. To determine whether the Salp15 gene is expressed in *Ixodes persulcatus* and *Ixodes sinensis*, principle vectors of Lyme borreliosis in China, we studied transcriptions of this gene in semi-engorged larvae, nymph and adults of these two species. A total of eight Salp15 homologues, five in *I. persulcatus* and three in *I. sinensis*, were identified by reverse transcriptase–polymerase chain reaction (RT-PCR). Interestingly, the intra-species similarity of Salp15 is approximately equal to its interspecies similarity and more than one Salp15 protein is expressed in a certain tick developmental stage. Comparison of DNA and proteins with other available tick Salp15 homologues suggests that the Salp15 superfamily is genetically conserved and diverse in the *Ixodes ricinus* complex. These findings indicate that Salp15 proteins in the *I. ricinus* complex may play an essential role in interacting with the host immune system and transmission of *Borrelia* genospecies.

## Introduction

Ticks are obligate arthropod exoparasites that can transfer pathogens to their hosts via their saliva. Tick saliva contains a variety of physiologically active molecules, which are vital not only for effective attachment and engorgement [1], [2] but also for pathogen transmission and host immunological regulations. Serotonin, (a powerful and long-lasting vasoconstrictor)[3], TAP (a tick anticoagulant peptide) [4], [5] and IgBP, a kind of tick defensin that binds imbibed vertebrate IgG that has passed through the tick's gut barrier into the hemolymph [6], [7], [8], are all known to facilitate tick attachment and engorgement. In addition, a variety of Salp proteins (Salp14, Salp20 and Salp25D) and p11 have been found to be active in pathogen transmission, regulation of host immunological responses and pathogensis [9], [10], [11], [12], [13], [14], [15].

Lyme borreliosis, commonly known as Lyme disease, is a tick-borne disease caused by spirochetes of various *Borrelia* genospecies[16], including *B. burgdorferi sensu stricto* in the USA [17], *B. burgdorferi sensu stricto*, *B. garinii* and *B. afzelii* in Europe [18], and *B. garinii* and *B. afzelii* in China [19]. Almost all the known vectors of Lyme borreliosis are members of the *I. ricinus* complex. The most prevalent Ixodid species are *Ixodes scapularis* that occurs mainly in eastern North America, *Ixodes pacificus* found in western North America, *Ixodes ricinus*, a species abundant in Europe and *Ixodes persulcatus* from the Far East, including northern China. Of these, *Ixodes pacificus*, *I*.*ricinus, I.scapularis* and *I. persulcatu*s and *I. sinensis* are regarded as the most effective disease vectors [20], [21], [22], [23]. However, the disease transmission mechanisms of these tick species are not well understood.

Salp15, a 15-kDa tick salivary gland protein, adheres to the outer surface protein C of *Borrelia burgdorferi* spirochetes thereby protecting them from the mammalian immune system. Salp15 is known to be able to suppress host immunity through complicated mechanisms, such as binding to CD4 to inhibit CD4^+^ T cell activation, thwarting dendritic cell activities, and altering the expression level of cytokines. Because of these properties, scientists have suggested that Salp15 could be a potential target for the development of vaccines against Lyme spirochetes [24], [25], [26]. Recently, Salp15 homologues have been characterized from some species in the *Ixodes ricinus* complex in different parts of the world [14], [18], [27], [28]. However, no comparable information is available on Salp15 in *Ixodes persulcatus* and *I. sinensis*, two members of the *Ixodes ricinus* complex from China. We here present five Salp15 coding sequences from *I. persulcatus*, from northern China and three from *I. sinensis*, from southern China. We also describe the genetic diversity of putative Salp15 amino acid sequences among different species and developmental stages of the *I. ricinus* complex and discuss the possible functional significance of this variation.

## Materials and Methods

### Ethics statement

The study had received the specific approval of Institutional Animal Care and Use Committee (IACUC) of Beijing institute of Microbiology and Epidemiology. We were informed the objectives, requirements and procedures of the experiments. Before each feeding process, a single dose of a non-steroidal anti-inflammatory agent (NSAID) Aspirin was orally administrated to mice to alleviate suffering of mice following the guidance of IACUC of Beijing institute of Microbiology and Epidemiology.

### Ticks

Adult ticks of *Ixodes sinensis* were collected in Henan province during the winter of 2011 and adult ticks of *Ixodes persulcatus* in Heilingjiang province during the summer of 2000. Larvae were obtained from eggs laid by adults maintained in glass tubes. Approximately 50 larvae were placed on Kunming mice (SPF) for engorgement and the engorged ticks were subsequently transferred to glass tubes to molt under laboratory conditions as described previously [29].

### Purification of *I. sinensis* and *I. persulcatus* RNA and RT-PCR

Larvae, nymphs and adults of *Ixodes sinensis* and *Ixodes perculcatus* were allowed to feed on mice until semi-engorged and subsequently removed and ground in liquid nitrogen. RNA samples of tick salivary gland were extracted and purified according to the manufacturer's instruction using an RNeasy mini kit (QIAGEN Co. Ltd.). An aliquot of the total RNA (1.6 μg or 2 μg) was reverse-transcribed using an oligo-dT primer (20 pmol) in a reaction volume of 20 μl according to the manufacturer's instructions using an AMV First Strand cDNA Synthesis Kit (NEB Co. Ltd.). The specific primers Fsalp: 5′-ATGGAATCTTTCGTCGCAATG-3′ and Rsalp: 5′-CTAACATCCGGGAATGTGC-3′ were designed according to Salp15 CDS (coding sequence) from *I. scapularis* (AAK97817), *I. ricinus* (EU128526; ABU93614; ABU9361), *I. persulcatus* (BAH09310; BAH09311) and *I. pacificus* (ACV32166), which had been deposited either in GenBank or the European Bioinformatics Institute. These primers were used to amplify Salp15 homologues in three developmental stages (larva, nymph and adult) of *I. sinensis* and *I. persulcatus*. The reaction mixture for PCR contained 2 mM Mg^2+^, 0.2 mM of deoxynucleoside triphosphate (dNTPs), 1.25 U DNA polymerase (Takara Co. Ltd.), 20 μM of each primer and 4 ng cDNA as templates in a final 50 μl volume. The cycling conditions for PCR were 3 min pre-denaturation at 95°C, 35 cycles of denaturation at 94°C for 30 s, annealing at 53°C for 40 s, extension at 72°C for 1 min, and final extension at 72°C for 10 min followed by a hold step at 4°C. The amplified products were visualized under UV light with a ChampGel-3200 Photographic system. PCR amplification products were purified according to the manufacturer's protocol provided for the QIAquick Gel Extraction Kit (QIAGEN Co. Ltd.) To clone Salp15 coding sequences, purified cDNA fragments were ligated into the cloning vector using the *pEASY*- T1 Simple Cloning Vector kit (Transgen Co. Ltd.) and then transformed into the competent *E. coli* strain trans5α (Transgen Co. Ltd.). The purified positive plasmids were sequenced on an automatic DNA sequencer (ABI 3730) with M13 primers by the Sangon Bio-technique Company (Beijing, China).

### Sequence analysis

RNA sequences were analyzed using CLC Genomics Workbench software (CLC bio, Inc.). After diminishing clone vector sequence contamination, the alignment of Salp15–like sequences was performed using the ClustalX 2.1 program. The nucleotide and amino acid similarities of salp15 among different *Ixodes* species were calculated using the Needle software package (v6.0.1) in WebLab [30]. The signal peptide cleavage sites in amino acid sequences were predicted by Signal P 4.0 [31].

### Phylogentic analysis

Sequences were aligned using ClustalX 2.1 software [32]. An unrooted Neighbor-Joining tree was constructed with Mega 4.0 [33], [34]. The parameter setups were as follows: p-distance; bootstrap: 1,000 replications; and gap/missing data: complete deletion; model: the Jone-Thorton-Taylor substitution matrix [18], [35].

## Results

### I. persulcatus and I. sinensis Salp15 homologues

Five cDNA clones encoding Salp15 homologues were identified from semi-engorged *Ixodes persulcatus* by Blastp (http://blast.ncbi.nlm.nih.gov/). Three of these were from the larvae of *I. persulcatus*; Ipers-2(408 bp) (JX094824), Ipers-3(375 bp) (JX094825) and Ipers-4 (414 bp) (JX094826) respectively. One Salp15-like sequence was obtained from *I. persulcatus* nymphs; Ipers-1(393 bp) (JX094823), and one from adults: Ipers-5(414 bp) (JX500419). Another three Salp15 homologues were obtained from *I. sinensis*: *I. sinensis* Is-2(393 bp) (JX094822) from larvae, Is-3(396 bp) (JX500420) from nymphs, and Is-1(414 bp) (JX094821) from adults. The experiment to amplify Salp15 homologs was repeated three times and each product was sequenced and identified from at least ten clones in each experiment.

### Signal peptide in Salp15 N- terminal

**Figure 1:**
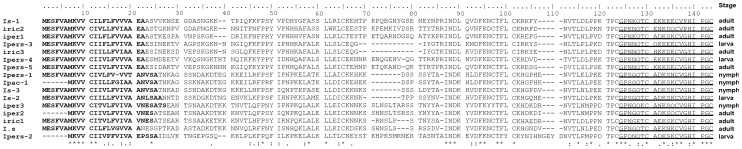
Comparison of Salp15 animo acid sequences from *Ixode persulcatus* and *Ixodes sinensis* with selected Salp15 sequences from other *Ixodes* species. *Footnote*: *I. persulcatus* Ipers-1 (JX500419), *I. persulcatus* Ipers-2 (JX094824), *I. persulcatus* Ipers-3 (JX094825), *I. persulcatus* Ipers-4(JX094826) from *I. persulcatus* (northern China), *I. sinensis* Is-1 (JX094821), *I. sinensis* Is-2 (JX094822), *I. sinensis* Is-3 (JX500420) from *I. sinensis* (southern China), *I. ricinus* iric1(ABU93613), *I. ricinus* iric2(ABU93614), *I. ricinus* iric3(ABU93615) from *I. ricinus* (Europe), *I. persulcatus* iper1(BAH09310), *I. persulcatus* iper2(BAH09311) from *I. persulcatus* in Japan, *I. persulcatus* iper3(ACV32167) from *I. persulcatus* (Russia), *I. pacificus* Ipac-1(ACV32166) from *I. pacificus* and *I. scapularis* Salp15(AAK97817) (North America). Positions of amino acid sequence identity, amino acid residue similarity and highly conserved amino acid substitutions are marked by an asterisk, full stop and colon, respectively. Signal Peptides of each sequence are in bold type. CD4 binding residues are underlined.

The known Salp15 sequences were predicted to contain a signal peptide in their N-terminus which would be cleaved during protein expression [18], [27], [28], [36] (Fig. 1). The eight homologues we obtained also shared this trait (Fig. 1), a feature which was confirmed by Signal P4.0 (http://www.cbs.dtu.dk/services/SignalP/). This predicted that the amino acid lengths of the signal peptides of Ipers-2, Ipers-1, Ipers-3, Ipers-4 from *I. persulcatus* were 24, 22, 22 and 22 respectively. For *I. sinensis*, the predicted signal peptide lengths of Is-3, Is-2 and Is-1 were 25, 26 and 22, respectively.

### Genetic diversity of Salp15 in the *I.ricinus* complex

We used the EBLOSUM62 matrix in the web-based software Needle (v6.0.1) from WebLab (http://weblab.cbi.pku.edu.cn), to align the putative proteins obtained with Salp15 homologs from other species in the *Ixodes ricinus* complex. Nucleic acid sequences were aligned with the EDNAFULL matrix [37]. A total of 16 Salp15 sequences were aligned and compared each other. The overall similarity values of amino acid sequences among the different sequences range from 53.1 to 94.4% (Tab. 1). The maximum similarity value between amino acid sequences is 94.4% between *I. persulcatus* Ipers-3 and *I. ricinus* iric3, followed by 89.1% between *I. persulcatus* iper-3 and *I. persulcatus* iper-2, then53.1% between *I. ricinus* iric2 and *I. persulcatus* Ipers-2. Overall nucleic acid similarities among the reference sequences ranged from 58.4 to 93.1% (Tab. 1). *I. ricinus* iric3 exhibits the maximum identity value of 93.1% with *I. persulcatus* Ipers-3, followed by 89.7% between *I. persulcatus* iper-3 and *I. persulcatus* iper-2 and finally 58.4% is between *I. ricinus* iric2 and *I. persulcatus* Ipers-2 (Table S1).

**Table 1:** Percent identity and similarity of Salp15 coding sequences among different *Ixodes* species.

		Amino acid level															
		larva			nymph	adult	nymph	adult	adult	larva	nymph	adult	adult			adult	nymph
		Ipers-2	Ipers-3	Ipers-4	Ipers-1	Ipers-5	iper3	iper1	iper2	Is-2	Is-3	Is-1	iric1	iric2	iric3	Salp15	Ipac-1
Nucleotide level	Ipers-2		*54.9%*	*55.9%*	*61.2%*	*59.3%*	*60.5%*	*56.0%*	*66.0%*	*60.8%*	*58.1%*	*55.2%*	*63.2%*	*53.1%*	*56.8%*	*57.6%*	*63.5%*
	Ipers-3	58.90%		*73.7%*	*63.4%*	*78.1%*	*57.1%*	*67.2%*	*55.0%*	*66.7%*	*63.3%*	*71.5%*	*61.8%*	*67.9%*	*94.4%*	*62.1%*	*59.7%*
	Ipers-4	60.60%	76.40%		*59.6%*	*81.0%*	*59.4%*	*67.6%*	*57.2%*	*63.4%*	*59.9%*	*69.6%*	*61.6%*	*72.5%*	*74.5%*	*62.3%*	*55.1%*
	Ipers-1	61.50%	66.40%	65.50%		*58.0%*	*75.4%*	*64.0%*	*71.7%*	*82.4%*	*87.8%*	*62.1%*	*79.1%*	*60.3%*	*64.9%*	*71.9%*	*87.8%*
	Ipers-5	57.80%	79.50%	84.00%	63.70%		*60.7%*	*75.4%*	*56.5%*	*62.1%*	*59.3%*	*73.9%*	*60.9%*	*71.7%*	*78.1%*	*65.8%*	*57.1%*
	iper3	59.90%	62.80%	66.90%	76.70%	66.90%		*60.4%*	*89.1%*	*75.4%*	*77.5%*	*58.9%*	*87.7%*	*61.2%*	*57.9%*	*79.7%*	*70.3%*
	iper1	64.00%	72.10%	76.80%	66.30%	78.00%	65.90%		*58.3%*	*61.9%*	*66.2%*	*78.7%*	*64.3%*	*77.9%*	*67.9%*	*66.7%*	*60.1%*
	iper2	69.40%	58.70%	61.40%	72.80%	63.20%	89.70%	62.70%		*73.9%*	*76.8%*	*61.4%*	*85.5%*	*61.4%*	*55.2%*	*80.4%*	*74.2%*
	Is-2	63.70%	64.50%	66.70%	86.90%	64.10%	77.90%	62.80%	75.40%		*82.4%*	*63.8%*	*79.1%*	*60.3%*	*65.6%*	*76.1%*	*79.4%*
	Is-3	63.40%	64.70%	67.90%	87.10%	64.30%	78.70%	63.90%	76.80%	87.50%		*60.4%*	*79.3%*	*63.3%*	*62.7%*	*75.6%*	*82.4%*
	Is-1	60.50%	72.00%	74.70%	63.60%	77.20%	63.60%	82.30%	60.40%	59.90%	61.00%		*60.3%*	*77.2%*	*71.5%*	*62.1%*	*55.7%*
	iric1	60.00%	64.70%	66.70%	77.80%	63.70%	88.80%	62.10%	62.70%	81.70%	80.20%	61.20%		*61.9%*	*60.6%*	*80.0%*	*73.9%*
	iric2	58.40%	70.40%	77.00%	62.10%	74.90%	66.50%	80.10%	58.40%	59.90%	61.10%	82.00%	61.00%		*69.3%*	*62.4%*	*56.3%*
	iric3	57.30%	93.10%	74.20%	62.80%	78.00%	61.10%	70.60%	59.20%	60.00%	61.90%	71.50%	63.10%	70.60%		*63.0%*	*60.7%*
	Salp15	62.90%	65.70%	67.10%	73.40%	66.50%	83.00%	65.70%	80.10%	78.30%	76.80%	64.00%	82.20%	63.00%	64.20%		*68.1%*
	Ipac-1	65.80%	62.10%	59.70%	89.40%	60.20%	69.10%	60.80%	75.00%	81.80%	81.60%	57.50%	73.10%	59.30%	58.80%	69.70%	

Ipers-1, Ipers-2, Ipers-3, Ipers-4, Ipers-5 are from *I. persulcatus* collected in northern China; iper1 and iper2 are from *I. persulcatus* collected in Japan; iper3 is from *I. persulcatus* collected in Russia; Is-1, Is-2 and Is-3 are from *I. sinensis* collected in southern China; iric1, iric2 and iric3 are from *I. ricinus* collected in Europe; Salp15 is from *I. scapularis* collected in eastern North America; Ipac-1 is from *I. pacificus* collected in western North America. Percentages in italics indicate the similarity between two sequences; Regular type indicates identity.

Similarity scores between the last 20 C- terminal amino acids are even higher; 95.0% between *I. persulcatus* Ipers-3 and *I.ricinus* iric3, 95.0% between *I. persulcatus* iper-3 and *I. persulcatus* iper-2 and 85.0% between *I. ricinus* iric2 and *I. persulcatus* Ipers-2. This suggests that this fragment is more conserved than the rest of Salp15 (Fig. 1). High variability in protein sequences was observed in the N- terminus of the approximate 140 deduced-amino-acid sequence, with three particularly hypervariable regions found between residues 21 to 42, 66 to 86, and 101 to 114 (Fig. 1).

**Figure 2:**
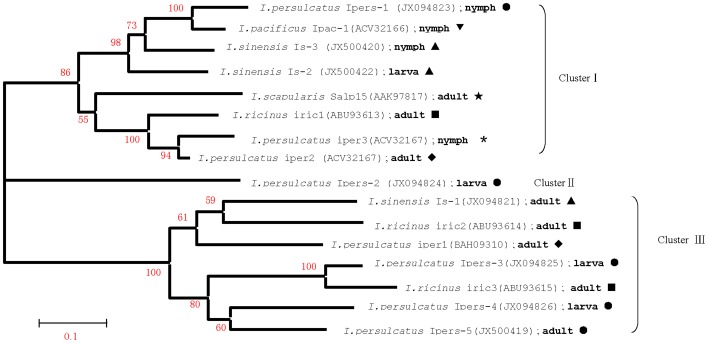
Phylogenetic tree of Salp15 homologues from members of *Ixodes ricinus* complex constructed with Mega 4 Tree Explorer. *Footnote*: The developmental stage of ticks from which sequences were obtained is in bold type. ▴ indicates Salp15 homologues from *I. sinensis* collected in southern China; • indicates the Salp15 homologues from *I. persulcatus* collected in northern China. *indicates the Salp15 homologues from *I. persulcatus* collected in Russia; ⧫ indicates Salp15 homologues from *I. persulcatus* collected in Japan; ▪ indicates Salp15 homologues from *I. ricinus* collected in Europe; ★ indicates the Salp15 from *I. scapularis* collected in easternNorth America. ▾ indicates the Salp15 from *I.pacificus* collected in western North America.

All bootstrap values in the phylogenetic tree constructed using Mega 4.0 exceed 50% (Fig. 2). Bootstrap values of each clade are as follows; *I. persulcatus* Ipers-3 and *I. ricinus* iric3 100%, *I. persulcatus* iper-3 and *I. persulcatus* iper-2 94%, *I. persulcatus* Ipers-1 and *I. pacificus* Ipac-1 100%. However, the phylogenetic tree shows that some sequences from the same *Ixodes* species also belong to different groups. For example, *I. ricinus* iric3 and *I. ricinus* iric2 are in cluster III, *I. ricinus* iric1 in cluster I, *I. persulcatus* iper1 from Japan in cluster III, *I. persulcatus* iper2 from Japan and *I. persulcatus* iper3 from Russia in cluster I, *I.sinensis* Is-1 from South China are in cluster III, *I.sinensis* Is-2 and *I.sinensis* Is-3 in cluster I. A similar pattern is apparent in the branch orders of *I. persulcatus* Salp15 homologues. Furthermore, branch orders are not related to developmental stage in certain *Ixode*s species. For example, *I. persulcatus* Ipers-3 and *I. persulcatus* Ipers-4 from larval stage fall into cluster III but *I. persulcatus* Ipers-2 belongs to cluster II. Interestingly, some sequences from the same developmental stage among different species belong to the same subgroup, for example, *I. sinensis* Is-1, *I. ricinus* iric2 and *I. persulcatus* iper1 from adults ticks belong to the same subgroup. Three nymph tick sequences, *I. persulcatus* Ipers-1, *I. pacificus* Ipac-1 and *I. sinensis* Is-3 also belong to the same subgroup.

## Discussion

Salp15 is a multi-functional protein that plays roles in the interface between both the host and vector and vector and pathogen. At the host-vector interface, Salp15 serves as a specific receptor of CD4 on host T cells, which can thwart the initiation of TCR (T cell receptor) ligation-induced signaling cascades, such as the activation of Lck (one src family protein kinase) upon TCR engagement, reduction of IL-2 production and the formation of lipid rafts and actin polymerization [38], [39], [40], [41]). The Salp15 sites directly associated with CD4 are the 20 C-terminal amino acids of which is a highly conservative fragment throughout the whole length of Salp15 among different Ixodid ticks [38], including the 5 Salp15 homologs from *I. persulcatus* and the 3 from *I. sinensis*. Theoretically, these 8 putative proteins could probably bind to CD4 and inhibit T-cell activation in a similar way. In addition, *I. scapularis* Salp15 can also interact with DC-SIGN on dendritic cells, which results in activation of the serine/threonine kinase Raf-1 and thus, suppresses human dendritic cell (DC) functions via the Raf-1/MEK-dependent signaling pathway [42]. However, whether the 8 *I. persulcatus* and *I. sinensis* Salp15 homologues do inhibit DC function requires further experimental verification.

At the vector-pathogen interface, *I. scapularis* Salp15 may directly adhere to the *B. burgdorferi* OspC, thereby helping this pathogen evade the host immune system [2], [24], [43]. Moreover, Salp15 iric1 from *I. ricinus* and its homologues from *I. scapularis* could protect *Borrelia burgdorferi sensu lato* against complement-mediated killing [26], [43]. It is noteworthy that Salp15 iric1 was protective to *Borrelia burgdorferi sensu strict* but provided no significant protection to *B. garinii* and *B. afzelii* [26]. We assume that these 8 Salp15 homologues protect distinct Lyme spirochetes in a similar manner and are therefore potential targets for preventing transmission of Lyme spirochetes. Identification of Salp15 homologs from Lyme disease vectors and comparison of these with known Salp15 members could lead to the discovery of function-conserved sites of Salp15 and thus the development of vaccines based on Salp15.

We identified the first Salp15 homologues from *I. persulcatus*, which is found in northern China, and *I. sinensis*, which is native to southern China. All 8 homologues contain the conservative domains previously identified in other members of the *I. ricinus* complex: *I. scapularis*, *I. ricinus*, *I. persulcatus* and *I. pacificus*, suggesting that they all belong to the Salp15 super-family (Fig. 2). However, the high level of sequence variation among homologues might indicate different functions or roles. This raises two major questions. The first is whether the observed diversity of Salp15 homologues occurs within single tick species? The second is if there a specific correlation between some transcripts of Salp15 homologues and tick developmental stages? In other words, whether some Salp15 proteins are only transcribed in a particular developmental stage is needed to determine.

To answer these questions, we performed a Position Specific Iterated Search (PSI-BLAST) on the NCBI website to identify proteins similar to Salp15 sequences [44], [45]. Evidence of a correlation between Salp15 homologues and developmental stages was limited due to the paucity of references available on Salp15 transcriptions in specific developmental stages of *I. ricinus* complex.Salp15 homologues display a high degree of both intra- and inter-species diversity. We tried to examine the Salp 15 copies in the genome of *Ixodes scapularis*, however, there are none completed *I. scapularis* genome information available currently. Most sequences are scaffolding or contigs with information lost. We also conducted an alternative investigation against *I. scapularis* expressed sequence tags (https://www.vectorbase.org/downloads?field_organism_taxonomy_tid=340&field_status_value=Current) substituting its genome. As showed in the supplement text (Text S1), only one copy Salp15 homologes is available i.e-I.s Salp15 we used in the present paper. Interestingly, we found that more than one Salp15 homologue is simultaneously transcribed in a particular developmental stage of *I. persulcatus* from China and Japan and in *I. ricinu*s from Europe (Since only one Salp15 homologue was available for *I. scapularis*, this species was not included in this investigation).

Because some Salp15 proteins are specific to a particular tick species, we assumed that these would have higher similarity in the same species compared to others in the *I. ricinus* complex. However, the results of our investigation did not support this hypothesis. For example, the five sequences from *I. persulcatus* have an intraspecies average amino acid similarity value only slightly lower than the interspecies average value *I. persulcatus* and Europe *I. ricinus* iric1 (being 64.5% to 65.3%). Similarly, the average value within *I. sinensis* is 68.8%, slightly lower than the 71.2% similarity between the Salp15 proteins of *I. sinensis* and *I. scapularis*.

Furthermore, the sequences transcribed in a particular developmental stage of the same species were no more similar than their counterparts transcribed in other developmental stages within same species. For instance, the average similarity of the sequences, Ipers-2, Ipers-3, and Ipers-4, transcribed in larvae of *I. persulcatus* was 61.5%, compared to 61.4% between three larval Salp15 and nymphal *I. persulcatus* Ipers-1 homologue,and 72.8% between three larval Salp15 and one adult *I. persulcatus* Ipers-5 homologue. Indeed, the three larval sequences were less similar to each other than they were to an adult sequence. The diversity of Salp15 shows no clear pattern with respect to either species or developmental stage.

This raises some other questions, such as what is the functional significance of the observed diversity of Salp15 proteins and why is the intraspecific variation in these nearly the same as the interspecific variation. One answer to the first question is that there may be more than one Salp15 genes in the tick genome that contribute to the diversity of Salp15 in the *Ixodes ricinus* complex. Another explanation may be that the apparent diversity is the result of alternative splicing processes of Salp15 precursor mRNA, which means that some Salp15 members are splice variants of others in a certain species.

There are two possible explanations for the second question. The first pertains to the period of *Ixodes* speciation. We postulate that the divergence time of each *Ixodes* species was relatively short allowing relatively little time for the evolution of Salp15 in newly formed sister species. Some Salp15 proteins are more similar to those in other species than they are to those in the same species. This may be an indication that they are the products of orthologous genes resulting from speciation events, in other words, that they are inherited from a common ancestor [45]. For example, *I. sinensis* Is-1 is probably an orthologous gene of *I. ricinus* iric2 and *I. ricinus* iric3 is probably an orthologous gene of *I. persulcatus* Ipers-3. On the other hand, there are some paralogous relationships between species resulting from gene duplication events in a particular lineage. For example, *I. persulcatus* Ipers-3, *I. persulcatus* Ipers-4 and *I. persulcatus* Ipers-5 are paralogous, as are *I. sinensis* Is-2 and *I. sinensis* Is-3 (Fig. 2). Another factor to bear in mind is the critical functions performed by Salp15 in these tick species. Salp15 directly interacts with some mammalian host immune system molecules thereby protecting ticks from the host's immunological defenses [43], [46]. The functions performed by Salp15 may be so vital that it has been highly conserved in tick evolution.

After analysis of Salp15 sequences, we also considered the possibility of whether the Salp15 homologues identified in *I. persulcatus* and *I. sinensis* are expressed throughout the entire life cycle. A low expression level of some Salp15 members may indicate that not all Salp15 sequences are amplified in a species in a particular developmental stage. However, contrary to the above hypothesis, this could also be the consequence of preferential expression patterns. We plan to address this question by using real-time RT-PCR to monitor changes in the expression pattern of *I. sinensis* and *I. persulcatus* Salp15 homolog in different developmental stages.

## Supporting Information

Table S1 **Percent identity of Salp15 coding sequences among different** ***Ixodes*** **species.** *Footnote*: Ipers-1, Ipers-2, Ipers-3, Ipers-4, Ipers-5 are from *I. persulcatus* collected in northern China; iper1 and iper2 are from *I. persulcatus* collected in Japan; iper3 is from *I. persulcatus* collected in Russia; Is-1, Is-2 and Is-3 are from *I. sinensis* collected in southern China; iric1, iric2 and iric3 are from *I. ricinus* collected in Europe; Salp15 is from *I. scapularis* collected in eastern North America; Ipac-1 is from *I. pacificus* collected in western North America.
(DOC)

Text S1 **Alignment of Salp15 coding sequences against** ***Ixodes scapularis*** **EST using Blastn program.**
(TXT)
