# Supplementary material for: Genetic Diversity of Salp15 in the Ixodes ricinus Complex (Acari: Ixodidae)
Source: PLoS One. 2014 Apr 8;9(4):e94131. doi: 10.1371/journal.pone.0094131 (PMC3979764; doi:10.1371/journal.pone.0094131)
Supplement: Table S1 — Percent identity of Salp15 coding sequences among different Ixodes species. Footnote: Ipers-1, Ipers-2, Ipers-3, Ipers-4, Ipers-5 are from I. persulcatus collected in northern China; iper1 and iper2 are from I. persulcatus collected in Japan; iper3 is from I. persulcatus collected in Russia; Is-1, Is-2 and Is-3 are from I. sinensis collected in southern China; iric1, iric2 and iric3 are from I. ricinus collected in Europe; Salp15 is from I. scapularis collected in eastern North America; Ipac-1 is from I. pacificus collected in western North America. (DOC) [file pone.0094131.s001.doc]

Table 1: Percent identity of Salp15 coding sequences among different *Ixodes* species

|  |  | Amino acid level | | | | | | | | | | | | | | | |
| --- | --- | --- | --- | --- | --- | --- | --- | --- | --- | --- | --- | --- | --- | --- | --- | --- | --- |
|  |  | larva | | | nymph | adult | nymph | adult | adult | larva | nymph | adult | adult | | | adult | nymph |
|  |  | Ipers-2 | Ipers-3 | Ipers-4 | Ipers-1 | Ipers-5 | iper3 | iper1 | iper2 | Is-2 | Is-3 | Is-1 | iric1 | iric2 | iric3 | Salp15 | Ipac-1 |
|  | Ipers-2 |  | 47.2% | 42.7% | 51.0% | 46.9% | 51.0% | 44.0% | 55.3% | 52.0% | 49.3% | 42.7% | 51.4% | 40.1% | 45.9% | 46.5% | 54.0% |
|  | Ipers-3 |  |  | 64.2% | 50.7% | 68.6% | 49.3% | 58.4% | 45.7% | 53.8% | 50.4% | 59.9% | 49.3% | 56.9% | 86.3% | 51.7% | 49.3% |
|  | Ipers-4 |  |  |  | 44.0% | 72.3% | 50.0% | 59.0% | 46.4% | 52.1% | 49.3% | 60.9% | 53.6% | 60.1% | 58.4% | 50.0% | 39.1% |
|  | Ipers-1 |  |  |  |  | 46.4% | 62.3% | 49.6% | 60.9% | 74.8% | 79.4% | 50.0% | 62.7% | 48.2% | 47.8% | 61.5% | 82.4% |
|  | Ipers-5 |  |  |  |  |  | 50.0% | 64.5% | 45.7% | 50.3% | 49.3% | 61.6% | 50.7% | 61.6% | 65.7% | 52.7% | 45.0% |
|  | iper3 |  |  |  |  |  |  | 50.4% | 84.8% | 60.1% | 65.9% | 50.0% | 79.0% | 50.4% | 47.1% | 68.8% | 55.1% |
|  | iper1 |  |  |  |  |  |  |  | 48.2% | 49.6% | 53.5% | 66.9% | 51.4% | 65.4% | 58.4% | 53.2% | 46.9% |
|  | iper2 |  |  |  |  |  |  |  |  | 63.0% | 67.4% | 49.3% | 76.8% | 47.9% | 44.1% | 66.7% | 62.1% |
|  | Is-2 |  |  |  |  |  |  |  |  |  | 77.1% | 51.1% | 65.7% | 48.2% | 51.9% | 62.3% | 71.0% |
|  | Is-3 |  |  |  |  |  |  |  |  |  |  | 49.0% | 66.4% | 51.1% | 50.0% | 65.9% | 72.5% |
|  | Is-1 |  |  |  |  |  |  |  |  |  |  |  | 50.4% | 67.6% | 57.7% | 51.7% | 45.0% |
|  | iric1 |  |  |  |  |  |  |  |  |  |  |  |  | 48.9% | 48.2% | 68.9% | 57.5% |
|  | iric2 |  |  |  |  |  |  |  |  |  |  |  |  |  | 56.2% | 49.6% | 45.1% |
|  | iric3 |  |  |  |  |  |  |  |  |  |  |  |  |  |  | 49.6% | 45.9% |
|  | Salp15 |  |  |  |  |  |  |  |  |  |  |  |  |  |  |  | 57.8% |
|  | Ipac-1 |  |  |  |  |  |  |  |  |  |  |  |  |  |  |  |  |

Table 1: Ipers-1, Ipers-2, Ipers-3, Ipers-4, Ipers-5 are from *I. persulcatus* collected in northern China; iper1 and iper2 are from *I. persulcatus* collected in Japan; iper3 is from *I. persulcatus* collected in Russia; Is-1, Is-2 and Is-3 are from *I. sinensis* collected in southern China; iric1, iric2 and iric3 are from *I. ricinus* collected in Europe; Salp15 is from *I. scapularis* collected in eastern North America; Ipac-1 is from *I. pacificus* collected in western North America.
